# Parameter estimation for stiff equations of biosystems using radial basis function networks

**DOI:** 10.1186/1471-2105-7-230

**Published:** 2006-04-27

**Authors:** Yoshiya Matsubara, Shinichi Kikuchi, Masahiro Sugimoto, Masaru Tomita

**Affiliations:** 1 Institute for Advanced Biosciences, Keio University, Fujisawa, Kanagawa, 252-8520, Japan; 2 Department of Bioinformatics, Mitsubishi Space Software Co. Ltd., Amagasaki, Hyogo, 661-0001, Japan

## Abstract

**Background:**

The modeling of dynamic systems requires estimating kinetic parameters from experimentally measured time-courses. Conventional global optimization methods used for parameter estimation, e.g. genetic algorithms (GA), consume enormous computational time because they require iterative numerical integrations for differential equations. When the target model is stiff, the computational time for reaching a solution increases further.

**Results:**

In an attempt to solve this problem, we explored a learning technique that uses radial basis function networks (RBFN) to achieve a parameter estimation for biochemical models. RBFN reduce the number of numerical integrations by replacing derivatives with slopes derived from the distribution of searching points. To introduce a slight search bias, we implemented additional data selection using a GA that searches data-sparse areas at low computational cost. In addition, we adopted logarithmic transformation that smoothes the fitness surface to obtain a solution simply. We conducted numerical experiments to validate our methods and compared the results with those obtained by GA. We found that the calculation time decreased by more than 50% and the convergence rate increased from 60% to 90%.

**Conclusion:**

In this work, our RBFN technique was effective for parameter optimization of stiff biochemical models.

## Background

The application of mathematical expressions for biochemical systems is useful for understanding complex biological phenomena. Reactions are expressed as rate equations, and the numerical integration of variables is applied to simulate these reactions. The simulation of biochemical models requires the identification of all kinetic parameters of each rate equation. However, almost all biochemical models, and large-scale models in particular, involve parameters that are extremely difficult to measure *in vivo *or *in vitro *[1-3] and the exclusive use of results from wet-bench experiments may be insufficient for the identification of all parameters. Therefore, to establish precise models, it is essential to determine unknown kinetic parameters from the time-courses of concentrations.

Various optimization algorithms such as the Levenberg-Marquardt method, genetic programming, simulated annealing, and evolutionary algorithms have been applied to infer parameters or equations in biochemical models [4-9]. The genetic algorithm (GA) [10] is commonly applied to these problems because of its global optimization ability [11-15].

However, the GA, including the real-coded GA [16], relies on stochastic optimization algorithms and requires vast numerical integrations to evaluate estimated parameters. In addition, the computational cost increases further when the target model has the stiffness that often appears in equations for biochemical systems because each solution requires small integration steps [17-19]. The application of the stochastic method to large-scale or stiff models involves enormously expensive computational time and superior hardware performance. In addition, use of GA elicits the sampling bias phenomenon [20,21]. This becomes an inherent problem that may result in failure in cases where an optimal parameter exists around the boundary of the parameter space. In particular, parameters of stiff systems tend to be allocated around the boundary of the search space because they involve different-order parameters.

The radial basis function network (RBFN) [22,23], a type of artificial neural network (ANN) [24], is one of the artificial learning methods that describe   complex non-linear relationships between inputs and outputs. The RBFN can solve parabolic, hyperbolic, and elliptic partial differential equations numerically [25] and is able to approximate non-linear time-courses effectively [26-28]. Due to the increased accumulation of biological information, RBFN are now applied in the biochemical field [29-33].

In the work presented here, we adopted RBFN to parameter estimation for local and global searches. RBFN enable to reduce the computational cost by omitting numerical integrations, resulting in solution of the above problems. In applying RBFN to biochemical modeling, we implemented subsidiary 2 improvements: (1) Since it is difficult to model highly nonlinear biochemical systems, we adopted a logarithmic transformation to both the input and output layer of the RBFN, thereby facilitating parameter-optimization. (2) RBFN require the selection of appropriate additional learning data to obtain a global minimum. Here we propose additional data selection using a GA for selecting additional learning data sets. This method adopts elemental GA to search data-sparse areas in the parameter space. We applied the proposed method to parameter estimation in a stiff biosystem. Our results demonstrate that compared to the GA, our method facilitates the acquisition of equivalent or more accurate parameters at half the calculation time and yields a 50% increase in the optimization success rate.

## Results

### Experimental conditions

**Figure 1:**
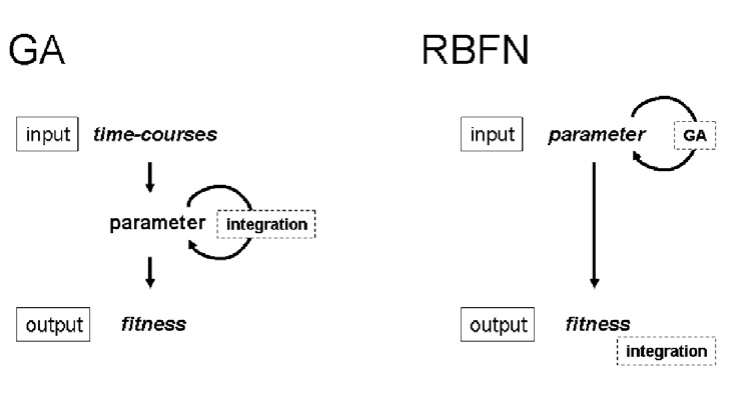
**Differences of learning procedures between GA and RBFN methods.**While GA learns the fitness between parameters and time-courses, RBFN learn the fitness of parameters. Therefore, numerical integrations used to evaluate the fitness of calculated time-courses are reduced from trial-and-error to one. The simple GA included in RBFN is used as an input data selection method of RBFN. RBFN enable to fast optimization by reducing the iterative calculations of numerical integrations.

**Figure 2:**
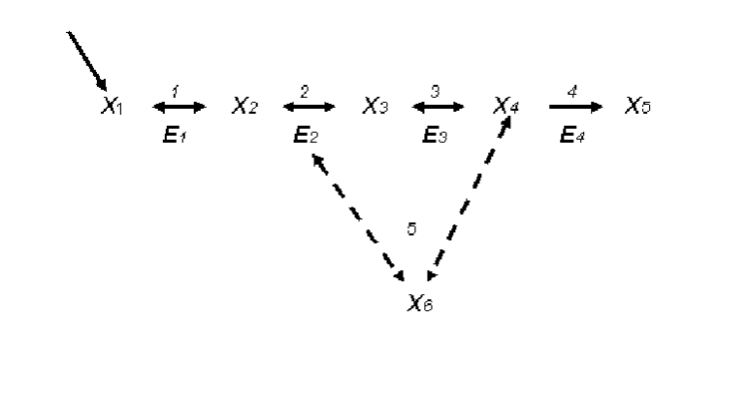
**Stiff model of the metabolic pathway used in this work**. It was composed of 5 reactions with 6 reactants, where *X*_5 _is the final product, *X*_1 _the external substance with fixed concentration, and *X*_2_, *X*_3_, *X*_4 _and *x*_6 _are intermediate metabolites, *e*_1_, *E*_2_, *E*_3_, and *E*_4 _are enzymes. The dotted line represents a feedback reaction. Details of the numbered reactions are shown in Table 1.

To examine the performance of the proposed method we used the metabolic pathway model shown in Figure 1. It is one of the typically stiff models employed to show the difficult optimization often observed in biochemical modeling [34-36]. The model is composed of 5 reactions with 6 reactants and 1 feedback loop in terms of enzyme concentration. We adopted a stiff model whose parameters differed by about 5 orders of magnitude since biochemical models often have stiffness and inference of those parameters is difficult. The kinetic equations and parameters used in the model are presented in Tables 1 and 2. The concentration of *X*_1 _is fixed as an external metabolite. Figure 2 shows calculated time-course data using Tables 1, 2, and 3; the concentrations of the enzymes are set to 0.01. From each time-course, 10 points were sampled for optimization (Time = 0.02, 0.04, 0.06, 0.08, 0.1, 0.2, 0.4, 0.6, 0.8 and 1.0). Table 4 shows the experimental conditions in this work. These parameters were selected empirically. The search ranges were *K_i _*∈ [0.1,1000]. The convergence indicates that learning attains a 10% relative error within 600 minutes. The processing time is the average of the calculation time required until learning succeeds. The test error is the relative error between given and calculated time-courses using initial conditions shown in Table 3. The computer environment was as follows: Pentium 4, Xeon 2 GHz, CPU with a memory size of 1024 MB. Our algorithm was implemented using Python language. The 4th-order Runge-Kutta method was employed as the ordinary differential equation solver; the time step was 0.0001.

### Performance of ADSGA

**Figure 3:**
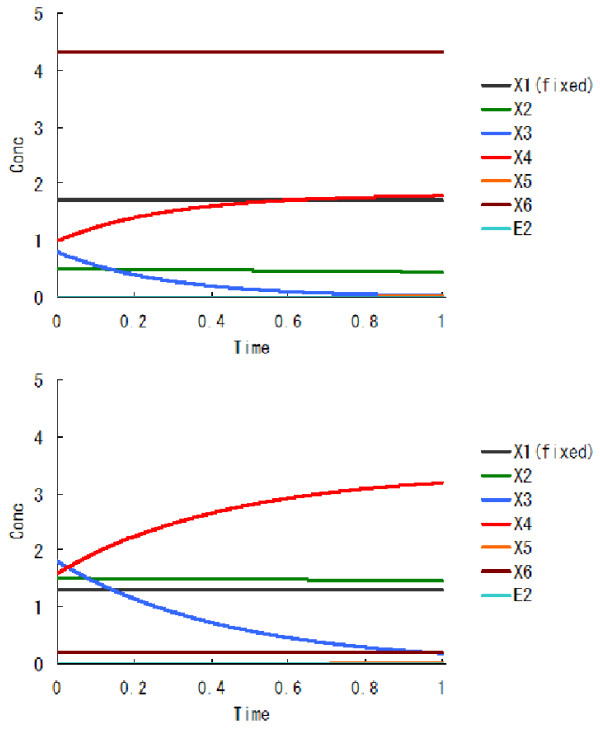
**Examples of time-courses that is artificially generated from the ordinary differential equations (Table 1) and the kinetic parameters (Table 2)**. The upper and lower graph indicate the cases No.3 and No.5 shown in Table 3, respectively. The changes of the time-courses of *X*_3 _and *X*_4 _are large compared with the other time-courses because of the stiffness. The *X*_1 _is the external substrate, and the value is not changed.

We compared the performance of additional learning using ADSGA with *k*NN. First, we inferred 2 parameters (*K*_11_, *K*_33_) in the Figure 2 model to examine the data distribution in the early learning phase. The additional learning of *k*NN and ADSGA was repeated 20 times. A density distribution of learned data is shown in Figure 3. Cases where *k *= 2, 4, 8, and 16 were examined. Deepening colors represent the density of existing data. For instance, a white grid represents no learning data in the parameter space; a black grid indicates that there are too many learning data in the parameter space. We found that the density distribution varied greatly depending on the value of *k*. A few grids did not include additional data in cases where *k *= 16. The grid that includes the answer exhibits the deepest color where *k *= 4, 8. In cases where *k *was not suitable, a few grids were over-searched while others were not selected appropriately. Therefore, *k*NN was not effective for finding the area that included few learned data. In cases where the parameter space is large, the additional data deviation is important because the distribution of the subsequent data influences learning in the early stage. In contrast, as indicated by the small color differences among the grids, ADSGA succeeded. There were minor deviations in density, indicating that additional learning data were selected from each grid almost equally. We also found that the grids near the correct answer were densely searched. This shows that global and local searches can be performed simultaneously using ADSGA.

Second, we inferred all parameters in the Figure 1 model. Table 6 shows that we succeeded in obtaining desirable results in terms of generalization ability using ADSGA and *k*NN with *k *= 4. *k*NN with the other *k *values attained lower achievements compared with our algorithm. Although ADSGA employed GA for determining additional data, the optimization speed was raised compared with *k*NN.

### Performance of logarithmic transformation

Table 6 shows the results using parameter transformation and fitness transformation in the inference of all parameters in the Figure 3 model. Log indicates the application of the logarithmic transformation of the fitness. The logarithmic transformation of the parameters applies to all cases. We found that the logarithmic transformation reduced the learning time by 23% while maintaining a high convergence rate. However, the test error increased by 16%. Optimization becomes simple with logarithmic transformation in cases where the view of the fitness space could be transformed to gently sloping. On the other hand, the test error increased slightly since it also changes the neighbor view of the answer. Since the parameters of power-law formalisms affect the parts of exponential factors, the perturbation leads to large changes in the variables and the individual fitness during their optimizations. The fitness values tend to become 0 in most of the search space since the results of numerical integrations become infinite. The smoothing of the error surface by the logarithmic transformation enables to simplify the search surface to optimize. The transformation achieved 1.2-fold faster optimization.

### RBFN application to stiff systems

The results obtained with the conventional GA method and our proposed RBFN method are presented side-by-side in the leftmost GA column and   the rightmost ADSGA+Log column in Table 6. We employed simplex crossover as the recombination procedure (SPX) [37]  as the recombination procedure and ranking selection as the selection procedure. Parameter transformation applies to all cases. Parameters for GA are shown in Table 5. These parameters were selected empirically.

The calculation time was reduced to less than half compared with GA. The 1.6-fold and 2.1-fold increases in the optimization speed are ascribable to RBFN and the combination of RBFN and the logarithmic transformation, respectively. RBFN learning omit the calculation of numerical integration because it learns the relationship between a parameter and its evaluation space. When problems are examined that require a long calculation time for each integration, the difference in the processing time between RBFN and GA increases further.

The convergence rate of RBFN was raised from 60% to 90% compared with that of GA. The RBFN application, not 2 subsidiary improvements, achieved to raise the convergence rate. Ref [20] reported a peculiar problem that pertains to the sampling bias phenomenon when the optimal solution exists at the edge of a parameter space. GA was unable to find the optimal solution because some parameters existed at the edge of a parameter space. As RBFN are only biased to the area near the estimated optimal solution, the optimal solution can be searched regardless of its location in a parameter space. The test error of RBFN did not decrease compared with that of GA. RBFN, which employ logarithmic transformation of fitness, are slightly inferior to GA in terms of local searches, resulting in a decline in the generalization ability compared with GA. If the logarithmic transformation is not applied in RBFN, the generalization ability of the 2 algorithms is equivalent and high convergence and fast optimization are retained.

## Discussion

GA involves the sampling bias phenomenon because the search region of unimodal normal-distribution crossover [38], parent-centric crossover [39], or SPX is biased to the interior of a parameter space. The phenomenon is bound to occur more frequently as the parameters become larger. To solve this problem, extrapolation-directed crossover [40] has been proposed; it is biased toward extrapolative crossover. In addition, boundary extension by mirroring [41] and toroidal search space conversion [42] have been proposed; they extend the search space arbitrarily. However, these techniques raise computational costs rather than conquering the sampling bias phenomenon.

The performance of RBFN was superior to that of GA in the parameter inference of stiff systems since it enables to reduce the number of numerical integrations.

For further improvement, the optimization algorithm is switched to the gradient method which is deterministic and can search local minima quickly at the end of the learning stage. A decrease in test error can be expected when a combination of algorithms is applied. It is considered that the additional improvement by the numerical integration methods such as Gear algorithm [43] is harmonious with our parameter estimation algorithm. The hybrid method is also one of tasks to be confirmed in the future. In this paper, we tested the case of stiff equations, and it is considered that non-stiff equations could also show the advantage of our method because of the simple dynamics.

We obtained solutions with low generalization ability when only a few time-course sets were given as input data. Predicted solutions with high generalization ability are likely to be biologically meaningful parameters. Careful selection of the number of sampling points [44] and application of the data smoothing method [19,45] are necessary when our method is applied to real problems because the most appropriate combination with our method is dependent on the data and the system in question.

RBFN are optimized by adding a new function *O*(*p*^2^) and then adding a new data set *O*(*m*^2^*+ mp + p*^2^), where *p *is the number of learned data and *m *is the number of the kernel function. The inference of numerous parameters causes a serious problem. Biological restriction as well as the deletion of several learning data in overcrowded areas can lead to a reduction in computational costs. In addition, problem decomposition strategies [15,46] are useful to reduce the number of parameters that should be inferred simultaneously.

## Conclusion

We applied RBFN to the parameter estimation of biochemical models, especially stiff systems. RBFN method could reduce the number of numerical integrations. Especially, it leaded to the fast optimization in stiff systems of biochmical models. It yielded equivalent or more accurate results compared to the parameters obtained with GA at half the calculation time and a 50% increase in the optimization success rate. Also we adopted 2 secondary learning techniques to RBFN. One is the fitness transformation that changes a fitness function into a gently sloping function and reduces the calculation time 0.8-fold. The other is the new selection method of learning data, ADSGA. ADSGA was able to quickly obtain optimal solutions with high generalization ability, almost equivalent to *k*NN with optimal *k*. In this paper, it was shown that our RBFN technique attained the parameter optimization for stiff biochemical models from measured time-courses only.

## Methods

### Overview of RBFN optimization method

While the RBFN are our main optimization algorithm to infer kinetic parameters, GA is used as a selection method for additional data for the RBFN. Figure 1 is an illustration of optimization using GA or RBFN. RBFN predict the optimal parameters by learning the relationship between parameters and fitness values. Unlike stochastic algorithms such as GA, this method replaces numerical integrations with slopes in the evaluation phase, thus numerical integration is significantly reduced. Our method is described as follows:

(a) Initialization

Parameters are generated and uniform random numbers are assigned to each parameter within the search space.

(b) Evaluation

Each parameter is set to the kinetic model to calculate the relative squared errors *E *between calculated and given time-courses. The fitness *F *is the reciprocal of *E, *i.e. *F = *1/E). If *F *is high, the calculated time-course resembles the given time-course. The goal is to search for the parameter space that maximizes *F*.

E=1lnm∑s=1l∑i=1n∑t=1T(Xcalci,s(t)−Xgiveni,s(t)Xgiveni,s(t))2,     (1)
 MathType@MTEF@5@5@+=feaafiart1ev1aaatCvAUfKttLearuWrP9MDH5MBPbIqV92AaeXatLxBI9gBaebbnrfifHhDYfgasaacH8akY=wiFfYdH8Gipec8Eeeu0xXdbba9frFj0=OqFfea0dXdd9vqai=hGuQ8kuc9pgc9s8qqaq=dirpe0xb9q8qiLsFr0=vr0=vr0dc8meaabaqaciaacaGaaeqabaqabeGadaaakeaacqWGfbqrcqGH9aqpdaWcaaqaaiabigdaXaqaaGqaciab=XgaSjab=5gaUjab=1gaTbaadaaeWbqaamaaqahabaWaaabCaeaadaqadaqaamaalaaabaGaemiwaG1aa0baaSqaaiabdogaJjabdggaHjabdYgaSjabdogaJbqaaiabdMgaPjabcYcaSiabdohaZbaakiabcIcaOiabdsha0jabcMcaPiabgkHiTiabdIfaynaaDaaaleaacqWGNbWzcqWGPbqAcqWG2bGDcqWGLbqzcqWGUbGBaeaacqWGPbqAcqGGSaalcqWGZbWCaaGccqGGOaakcqWG0baDcqGGPaqkaeaacqWGybawdaqhaaWcbaGaem4zaCMaemyAaKMaemODayNaemyzauMaemOBa4gabaGaemyAaKMaeiilaWIaem4CamhaaOGaeiikaGIaemiDaqNaeiykaKcaaaGaayjkaiaawMcaamaaCaaaleqabaGaeGOmaidaaOGaeiilaWIaaCzcaiaaxMaacqGGOaakcqaIXaqmcqGGPaqkaSqaaiabdsha0jabg2da9iabigdaXaqaaiabdsfaubqdcqGHris5aaWcbaGaemyAaKMaeyypa0JaeGymaedabaGaemOBa4ganiabggHiLdaaleaacqWGZbWCcqGH9aqpcqaIXaqmaeaacqWGSbaBa0GaeyyeIuoaaaa@7CFB@

where *l *is the number of time-course sets, *n *the number of experimentally given state variables, Xcalci(t)
 MathType@MTEF@5@5@+=feaafiart1ev1aaatCvAUfKttLearuWrP9MDH5MBPbIqV92AaeXatLxBI9gBaebbnrfifHhDYfgasaacH8akY=wiFfYdH8Gipec8Eeeu0xXdbba9frFj0=OqFfea0dXdd9vqai=hGuQ8kuc9pgc9s8qqaq=dirpe0xb9q8qiLsFr0=vr0=vr0dc8meaabaqaciaacaGaaeqabaqabeGadaaakeaacqWGybawdaqhaaWcbaGaem4yamMaemyyaeMaemiBaWMaem4yamgabaGaemyAaKgaaOGaeiikaGIaemiDaqNaeiykaKcaaa@37E4@ the numerically calculated concentration at time *t *of a state variable *X_i_*, and Xgiveni(t)
 MathType@MTEF@5@5@+=feaafiart1ev1aaatCvAUfKttLearuWrP9MDH5MBPbIqV92AaeXatLxBI9gBaebbnrfifHhDYfgasaacH8akY=wiFfYdH8Gipec8Eeeu0xXdbba9frFj0=OqFfea0dXdd9vqai=hGuQ8kuc9pgc9s8qqaq=dirpe0xb9q8qiLsFr0=vr0=vr0dc8meaabaqaciaacaGaaeqabaqabeGadaaakeaacqWGybawdaqhaaWcbaGaem4zaCMaemyAaKMaemODayNaemyzauMaemOBa4gabaGaemyAaKgaaOGaeiikaGIaemiDaqNaeiykaKcaaa@3979@ represents the given concentration at time *t *of a state variable *X_i_*. For expedience, when Xgiveni(t)
 MathType@MTEF@5@5@+=feaafiart1ev1aaatCvAUfKttLearuWrP9MDH5MBPbIqV92AaeXatLxBI9gBaebbnrfifHhDYfgasaacH8akY=wiFfYdH8Gipec8Eeeu0xXdbba9frFj0=OqFfea0dXdd9vqai=hGuQ8kuc9pgc9s8qqaq=dirpe0xb9q8qiLsFr0=vr0=vr0dc8meaabaqaciaacaGaaeqabaqabeGadaaakeaacqWGybawdaqhaaWcbaGaem4zaCMaemyAaKMaemODayNaemyzauMaemOBa4gabaGaemyAaKgaaOGaeiikaGIaemiDaqNaeiykaKcaaa@3979@ is 0, the denominator of *E *is set to 0.1.

(c) RBFN learning

RBFN learn the relationship between parameters and fitness values. The output of RBFN f(x) defines the view of the fitness space in the parameter estimation. The value is calculated from

f(x)=∑j=1mwjhj(x),     (2)
 MathType@MTEF@5@5@+=feaafiart1ev1aaatCvAUfKttLearuWrP9MDH5MBPbIqV92AaeXatLxBI9gBaebbnrfifHhDYfgasaacH8akY=wiFfYdH8Gipec8Eeeu0xXdbba9frFj0=OqFfea0dXdd9vqai=hGuQ8kuc9pgc9s8qqaq=dirpe0xb9q8qiLsFr0=vr0=vr0dc8meaabaqaciaacaGaaeqabaqabeGadaaakeaacqWGMbGzcqGGOaakcqWG4baEcqGGPaqkcqGH9aqpdaaeWbqaaiabdEha3naaBaaaleaacqWGQbGAaeqaaOGaemiAaG2aaSbaaSqaaiabdQgaQbqabaGccqGGOaakcqWG4baEcqGGPaqkcqGGSaalcaWLjaGaaCzcaiabcIcaOiabikdaYiabcMcaPaWcbaGaemOAaOMaeyypa0JaeGymaedabaGaemyBa0ganiabggHiLdaaaa@4713@

where *w_j _*is the *j*th weight, *x* the learning data from parameter space,   *m* the number of learning data,  and *h*_*j *_is the *j*th kernel function, e.g.,

hj(x)=exp⁡(−|x−cj|2r2),     (3)
 MathType@MTEF@5@5@+=feaafiart1ev1aaatCvAUfKttLearuWrP9MDH5MBPbIqV92AaeXatLxBI9gBaebbnrfifHhDYfgasaacH8akY=wiFfYdH8Gipec8Eeeu0xXdbba9frFj0=OqFfea0dXdd9vqai=hGuQ8kuc9pgc9s8qqaq=dirpe0xb9q8qiLsFr0=vr0=vr0dc8meaabaqaciaacaGaaeqabaqabeGadaaakeaacqWGObaAdaWgaaWcbaGaemOAaOgabeaakiabcIcaOiabdIha4jabcMcaPiabg2da9iGbcwgaLjabcIha4jabcchaWnaabmaabaWaaSaaaeaacqGHsislcqGG8baFcqWG4baEcqGHsislcqWGJbWydaWgaaWcbaGaemOAaOgabeaakiabcYha8naaCaaaleqabaGaeGOmaidaaaGcbaGaemOCai3aaWbaaSqabeaacqaIYaGmaaaaaaGccaGLOaGaayzkaaGaeiilaWIaaCzcaiaaxMaacqGGOaakcqaIZaWmcqGGPaqkaaa@4B54@

where *c_j _*is the center of the *j*-th kernel and *r *is the radial of kernels. The optimal weight of each kernel is immediately obtained by the matrix operation. Various methods determining internal parameters of RBFN, e.g. generalized cross-validation [47] or calculation of the marginal likelihood of the data [48], can adjust the centers of kernels [49]. In this study, we assume that the inputs of the data set are used as the fixed centers since adjustment involves high computational costs. For details of the supervised training scheme, refer to [50].

(d) Capturing the RBFN shape by GA

GA is used to search for the optimal parameters for the maximum *F *based on the function of parameters (input) and the fitness value (output) from the previous step. We employed real-coded GA; it could attain a high convergence success rate, fast optimization, and high accuracy compared with conventional binary GA [16]. We then adopted the GA for searching the optimal solution of the network at the present stage. The calculation cost of this procedure is very small since the GA does not require numerical integration in the evaluation phase.

(e) Additional data selection

If the solution obtained in the previous phase is not satisfactory, additional learning parameters are used to improve fitness. Two types of learning parameters are employed for relearning [51]: One is selected from the neighborhood of the current optimal parameters to add local information near the optimal point, the other is selected from a sparse area in the parameter space to add global information. Iterative learning using 2 types of data enables a local and global search for the optimal parameters.

(f) Repeat (b)-(e)

If the current optimal solution is unsatisfactory, learning is repeated from (b).

### Subsidiary improvements

#### Additional learning

RBFN capture the function of parameters and the fitness value by exercising feed-forward control of the optimal parameters. Additional learning is performed to search parameters with higher fitness. Parameters for learning are carefully selected from 2 areas in order to consider global and local optimizations. In one area learning data are few because a whole view of fitness function can be obtained by adding such data. The k-nearest-neighbor method (*k*NN) [52,53] is a clustering technique to find the sparse area. The density function of a data set *x*_*q *_is described as *f*(*x*_*q*_) = ∑y∈Dk
 MathType@MTEF@5@5@+=feaafiart1ev1aaatCvAUfKttLearuWrP9MDH5MBPbIqV92AaeXatLxBI9gBaebbnrfifHhDYfgasaacH8akY=wiFfYdH8Gipec8Eeeu0xXdbba9frFj0=OqFfea0dXdd9vqai=hGuQ8kuc9pgc9s8qqaq=dirpe0xb9q8qiLsFr0=vr0=vr0dc8meaabaqaciaacaGaaeqabaqabeGadaaakeaacqGHris5daqhaaWcbaGaemyEaKNaeyicI4mcbeGae8hraqeabaGaem4AaSgaaaaa@33F2@ dist(*x_q_*, *y*), where *y *is a set of *k *data near *x*_*q *_in all the data **D**, and dist(*x*_*q*_,*y*) represents the Euclidean distance of *x*_*q *_and *y. k*NN defines *x*, which has the maximum value of *f*(*x*_*q*_), as the most sparse data set. Optimal *k *changes with the distribution of data learned early, the dimension of the parameter space, and the number of groups or units of data. Therefore, searching the optimal parameters involves the computational costs for obtaining the optimal *k*.

We propose a method for selecting additional data for RBFN learning and capturing the rough surface using elemental GA. Note that GA was not applied to the parameter-estimation phase. We employ the simple GA to accelerate finding the next data. This is indicated in paragraph (d) of the previous section. This procedure is called additional data selection using GA (ADSGA). The rule for selecting data in ADSGA is as follows. First, the density function of the learned data is created by RBFN, where [1 1 ··· 1]^⊤ ^is applied as *F*. Next, the minimum value of the function is searched by simply using GA. ADSGA searches the minimum value of the function as the sparsest data set. Since ADSGA does not require control parameters such as *k*, it can create the density function without pilot studies.

The other additional learning data are selected from the area near the current optimal solution to obtain more detailed information. The size of this neighborhood is controlled by X,=rand(X^−L,X^+L)
 MathType@MTEF@5@5@+=feaafiart1ev1aaatCvAUfKttLearuWrP9MDH5MBPbIqV92AaeXatLxBI9gBaebbnrfifHhDYfgasaacH8akY=wiFfYdH8Gipec8Eeeu0xXdbba9frFj0=OqFfea0dXdd9vqai=hGuQ8kuc9pgc9s8qqaq=dirpe0xb9q8qiLsFr0=vr0=vr0dc8meaabaqaciaacaGaaeqabaqabeGadaaakeaadaWfGaqaaGqaciab=HfaybWcbeqaaiab=XcaSaaakiabg2da9Gqaaiab+jhaYjab+fgaHjab+5gaUjab+rgaKjabcIcaOiqbdIfayzaajaGaeyOeI0IaemitaWKaeiilaWIafmiwaGLbaKaacqGHRaWkcqWGmbatcqGGPaqkaaa@3EBA@, where X,
 MathType@MTEF@5@5@+=feaafiart1ev1aaatCvAUfKttLearuWrP9MDH5MBPbIqV92AaeXatLxBI9gBaebbnrfifHhDYfgasaacH8akY=wiFfYdH8Gipec8Eeeu0xXdbba9frFj0=OqFfea0dXdd9vqai=hGuQ8kuc9pgc9s8qqaq=dirpe0xb9q8qiLsFr0=vr0=vr0dc8meaabaqaciaacaGaaeqabaqabeGadaaakeaadaWfGaqaaGqaciab=HfaybWcbeqaaiab=XcaSaaaaaa@2F12@ represents the parameters for learning in the next repetition, X^
 MathType@MTEF@5@5@+=feaafiart1ev1aaatCvAUfKttLearuWrP9MDH5MBPbIqV92AaeXatLxBI9gBaebbnrfifHhDYfgasaacH8akY=wiFfYdH8Gipec8Eeeu0xXdbba9frFj0=OqFfea0dXdd9vqai=hGuQ8kuc9pgc9s8qqaq=dirpe0xb9q8qiLsFr0=vr0=vr0dc8meaabaqaciaacaGaaeqabaqabeGadaaakeaacuWGybawgaqcaaaa@2DF5@ is the current optimal solution, and rand(*A, B*) generates a vector consisting of uniform random numbers ∈ [*A, B*]. When the estimated optimal solution varies greatly with every learning session, the search continues to be defined as an early stage. When the solution seldom changes even with repeated learning sessions, the search is defined as a finishing stage. We recommend decreasing the *L *value as the search advances.

Two conditions are then defined to change the value of *L*: (1) the current estimated optimal solution X^
 MathType@MTEF@5@5@+=feaafiart1ev1aaatCvAUfKttLearuWrP9MDH5MBPbIqV92AaeXatLxBI9gBaebbnrfifHhDYfgasaacH8akY=wiFfYdH8Gipec8Eeeu0xXdbba9frFj0=OqFfea0dXdd9vqai=hGuQ8kuc9pgc9s8qqaq=dirpe0xb9q8qiLsFr0=vr0=vr0dc8meaabaqaciaacaGaaeqabaqabeGadaaakeaacuWGybawgaqcaaaa@2DF5@ is near the preceding estimated optimal solution X,
 MathType@MTEF@5@5@+=feaafiart1ev1aaatCvAUfKttLearuWrP9MDH5MBPbIqV92AaeXatLxBI9gBaebbnrfifHhDYfgasaacH8akY=wiFfYdH8Gipec8Eeeu0xXdbba9frFj0=OqFfea0dXdd9vqai=hGuQ8kuc9pgc9s8qqaq=dirpe0xb9q8qiLsFr0=vr0=vr0dc8meaabaqaciaacaGaaeqabaqabeGadaaakeaadaWfGaqaaGqaciab=HfaybWcbeqaaiab=XcaSaaaaaa@2F12@, and (2) the fitness of X^
 MathType@MTEF@5@5@+=feaafiart1ev1aaatCvAUfKttLearuWrP9MDH5MBPbIqV92AaeXatLxBI9gBaebbnrfifHhDYfgasaacH8akY=wiFfYdH8Gipec8Eeeu0xXdbba9frFj0=OqFfea0dXdd9vqai=hGuQ8kuc9pgc9s8qqaq=dirpe0xb9q8qiLsFr0=vr0=vr0dc8meaabaqaciaacaGaaeqabaqabeGadaaakeaacuWGybawgaqcaaaa@2DF5@ is better than that of X,
 MathType@MTEF@5@5@+=feaafiart1ev1aaatCvAUfKttLearuWrP9MDH5MBPbIqV92AaeXatLxBI9gBaebbnrfifHhDYfgasaacH8akY=wiFfYdH8Gipec8Eeeu0xXdbba9frFj0=OqFfea0dXdd9vqai=hGuQ8kuc9pgc9s8qqaq=dirpe0xb9q8qiLsFr0=vr0=vr0dc8meaabaqaciaacaGaaeqabaqabeGadaaakeaadaWfGaqaaGqaciab=HfaybWcbeqaaiab=XcaSaaaaaa@2F12@. If conditions (1) and (2) are fulfilled, *L *– α is substituted for the value of *L*. If only condition (1) is fulfilled, *L*_0_, which is the initial value of *L*, is substituted for the value of *L*. If neither (1) nor (2) is fulfilled, the value of *L *remains unchanged.

The simultaneous inclusion of global and local data can prevent convergence into a local minimum solution, and can obtain a better solution near the current optimal solution.

#### Logarithmic transformation

**Figure 5:**
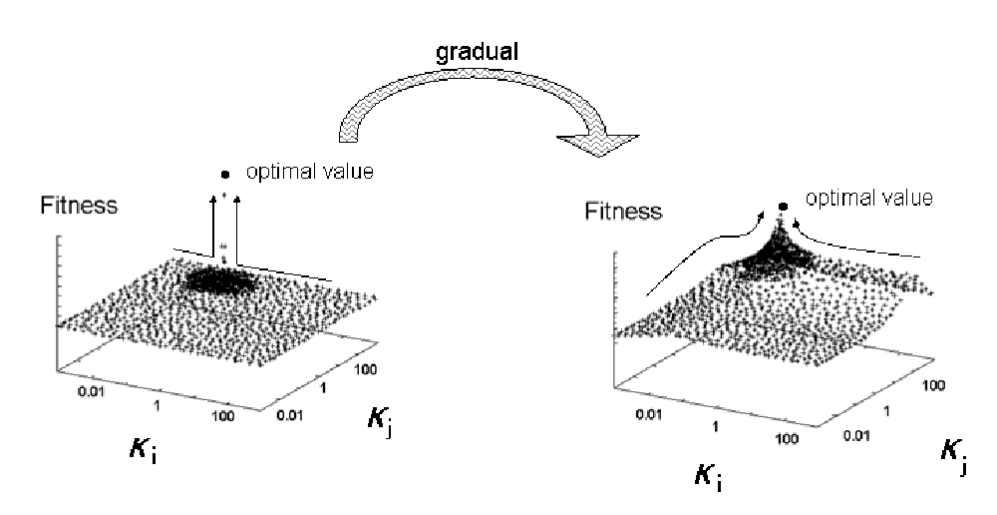
**The adoption of fitness transformation that facilitates parameter estimation using RBFN**. View of the fitness functions when 2 parameters (*K*_*i*_, *K*_*j*_) are estimated, where the X-axis and Y-axis represent logarithmic coordinates of data and the Z-axis represents fitness of the coordinates. On the right, logarithmic transformation of fitness is applied.

We applied parameter transformation and fitness transformation. Parameters in a metabolic pathway, especially a stiff system, may involve various scales. Such parameter spaces take the ridge structure [54] where the valley for optimal solutions exists in a very narrow space with respect to comparatively small scales. Ridge structures complicate the process of optimizing functions. Because parameter values are positive and fitness seldom changes with large parameter values, we adopted parameter transformation where a parameter space is mapped into a logarithmic space. The method enables dense searches for small-range parameters and avoids ridge structures, allowing optimization to progress smoothly. Fitness function is often a rapid-peaked function where values change rapidly near a certain point since biochemical models often exhibit strong nonlinearity. If gradient-descent methods such as ANN are used for searching an optimal solution in a rapid-peaked function, the optimal solution can be approached only from a data set near the solution. In such cases, many iterations are usually required to produce a solution. To overcome this problem, we propose fitness transformation where fitness is mapped into a logarithmic space. As a result, the function turns into a gently sloping function, so that the approach from nearby data becomes easier, with fewer iterations. A view of the fitness function is presented in Figure 5.

## Authors' contributions

YM implemented and evaluated the RBFN algorithm, and drafted the manuscript. SK made instructive contributions and designed the original specifications. MS provided valuable insights into optimization methods, and was involved in revising the implementation and manuscript. MT supervised the study in the overall design. All authors read and approved the final manuscript.

## Figures and Tables

**Figure 4:**
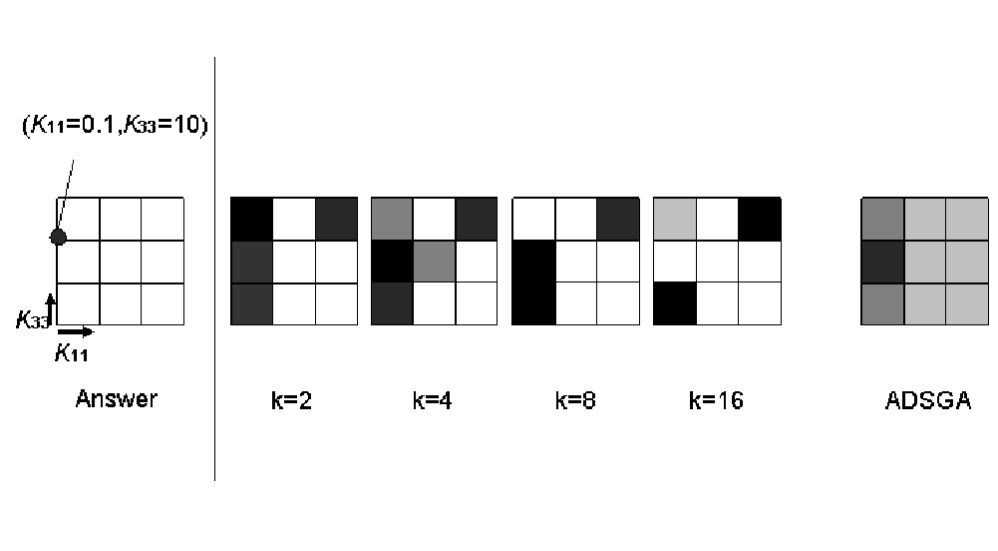
**The performance of ADSGA that is proposed for additional learning of RBFN**. From left to right: density distributions of learned data when 2 parameters (*K*_11_,*K*_33_) are estimated using *k*NN (*k *= 2, 4, 8, and 16) and ADSGA, where the X-axis and the Y-axis represent logarithmic coordinates of data. 'Answer' represents coordinates of the optimal solution. Deepening colors represent the increasing density of existing data. The Grids represent 0.1-100 from the left-bottom. This results show that ADSGA applied RBFN searches both data-sparse areas and answer-adjacent areas simultaneously.

**Table 1:** Kinetic equations used in the model. Reactions 1, 2, and 3 are reversible Henri-Michaelis-Menten reactions with one substrate and one product. Reaction 4 is an irreversible Henri-Michaelis-Menten reaction with one substrate and one product. Reaction 5 is a mass action reaction with 2 substrates and one product.

Reaction number	kinetic equation
1	d[X2]dt=(K11K12[X1]−K13K14[X2])[E1]K14[X2]+K12[X1]+K14K12−(K21K22[X2]−K23K24[X3])[E2]K24[X3]+K22[X2]+K24K22 MathType@MTEF@5@5@+=feaafiart1ev1aaatCvAUfKttLearuWrP9MDH5MBPbIqV92AaeXatLxBI9gBaebbnrfifHhDYfgasaacH8akY=wiFfYdH8Gipec8Eeeu0xXdbba9frFj0=OqFfea0dXdd9vqai=hGuQ8kuc9pgc9s8qqaq=dirpe0xb9q8qiLsFr0=vr0=vr0dc8meaabaqaciaacaGaaeqabaqabeGadaaakeaadaWcaaqaaiabdsgaKjabcUfaBjabdIfaynaaBaaaleaacqaIYaGmaeqaaOGaeiyxa0fabaGaemizaqMaemiDaqhaaiabg2da9maalaaabaGaeiikaGIaem4saS0aaSbaaSqaaiabigdaXiabigdaXaqabaGccqWGlbWsdaWgaaWcbaGaeGymaeJaeGOmaidabeaakiabcUfaBjabdIfaynaaBaaaleaacqaIXaqmaeqaaOGaeiyxa0LaeyOeI0Iaem4saS0aaSbaaSqaaiabigdaXiabiodaZaqabaGccqWGlbWsdaWgaaWcbaGaeGymaeJaeGinaqdabeaakiabcUfaBjabdIfaynaaBaaaleaacqaIYaGmaeqaaOGaeiyxa0LaeiykaKIaei4waSLaemyrau0aaSbaaSqaaiabigdaXaqabaGccqGGDbqxaeaacqWGlbWsdaWgaaWcbaGaeGymaeJaeGinaqdabeaakiabcUfaBjabdIfaynaaBaaaleaacqaIYaGmaeqaaOGaeiyxa0Laey4kaSIaem4saS0aaSbaaSqaaiabigdaXiabikdaYaqabaGccqGGBbWwcqWGybawdaWgaaWcbaGaeGymaedabeaakiabc2faDjabgUcaRiabdUealnaaBaaaleaacqaIXaqmcqaI0aanaeqaaOGaem4saS0aaSbaaSqaaiabigdaXiabikdaYaqabaaaaOGaeyOeI0YaaSaaaeaacqGGOaakcqWGlbWsdaWgaaWcbaGaeGOmaiJaeGymaedabeaakiabdUealnaaBaaaleaacqaIYaGmcqaIYaGmaeqaaOGaei4waSLaemiwaG1aaSbaaSqaaiabikdaYaqabaGccqGGDbqxcqGHsislcqWGlbWsdaWgaaWcbaGaeGOmaiJaeG4mamdabeaakiabdUealnaaBaaaleaacqaIYaGmcqaI0aanaeqaaOGaei4waSLaemiwaG1aaSbaaSqaaiabiodaZaqabaGccqGGDbqxcqGGPaqkcqGGBbWwcqWGfbqrdaWgaaWcbaGaeGOmaidabeaakiabc2faDbqaaiabdUealnaaBaaaleaacqaIYaGmcqaI0aanaeqaaOGaei4waSLaemiwaG1aaSbaaSqaaiabiodaZaqabaGccqGGDbqxcqGHRaWkcqWGlbWsdaWgaaWcbaGaeGOmaiJaeGOmaidabeaakiabcUfaBjabdIfaynaaBaaaleaacqaIYaGmaeqaaOGaeiyxa0Laey4kaSIaem4saS0aaSbaaSqaaiabikdaYiabisda0aqabaGccqWGlbWsdaWgaaWcbaGaeGOmaiJaeGOmaidabeaaaaaaaa@A493@
2	d[X3]dt=(K21K22[X2]−K23K24[X3])[E2]K24[X3]+K22[X2]+K24K22−(K31K32[X3]−K33K34[X4])[E3]K34[X4]+K32[X3]+K34K32 MathType@MTEF@5@5@+=feaafiart1ev1aaatCvAUfKttLearuWrP9MDH5MBPbIqV92AaeXatLxBI9gBaebbnrfifHhDYfgasaacH8akY=wiFfYdH8Gipec8Eeeu0xXdbba9frFj0=OqFfea0dXdd9vqai=hGuQ8kuc9pgc9s8qqaq=dirpe0xb9q8qiLsFr0=vr0=vr0dc8meaabaqaciaacaGaaeqabaqabeGadaaakeaadaWcaaqaaiabdsgaKjabcUfaBjabdIfaynaaBaaaleaacqaIZaWmaeqaaOGaeiyxa0fabaGaemizaqMaemiDaqhaaiabg2da9maalaaabaGaeiikaGIaem4saS0aaSbaaSqaaiabikdaYiabigdaXaqabaGccqWGlbWsdaWgaaWcbaGaeGOmaiJaeGOmaidabeaakiabcUfaBjabdIfaynaaBaaaleaacqaIYaGmaeqaaOGaeiyxa0LaeyOeI0Iaem4saS0aaSbaaSqaaiabikdaYiabiodaZaqabaGccqWGlbWsdaWgaaWcbaGaeGOmaiJaeGinaqdabeaakiabcUfaBjabdIfaynaaBaaaleaacqaIZaWmaeqaaOGaeiyxa0LaeiykaKIaei4waSLaemyrau0aaSbaaSqaaiabikdaYaqabaGccqGGDbqxaeaacqWGlbWsdaWgaaWcbaGaeGOmaiJaeGinaqdabeaakiabcUfaBjabdIfaynaaBaaaleaacqaIZaWmaeqaaOGaeiyxa0Laey4kaSIaem4saS0aaSbaaSqaaiabikdaYiabikdaYaqabaGccqGGBbWwcqWGybawdaWgaaWcbaGaeGOmaidabeaakiabc2faDjabgUcaRiabdUealnaaBaaaleaacqaIYaGmcqaI0aanaeqaaOGaem4saS0aaSbaaSqaaiabikdaYiabikdaYaqabaaaaOGaeyOeI0YaaSaaaeaacqGGOaakcqWGlbWsdaWgaaWcbaGaeG4mamJaeGymaedabeaakiabdUealnaaBaaaleaacqaIZaWmcqaIYaGmaeqaaOGaei4waSLaemiwaG1aaSbaaSqaaiabiodaZaqabaGccqGGDbqxcqGHsislcqWGlbWsdaWgaaWcbaGaeG4mamJaeG4mamdabeaakiabdUealnaaBaaaleaacqaIZaWmcqaI0aanaeqaaOGaei4waSLaemiwaG1aaSbaaSqaaiabisda0aqabaGccqGGDbqxcqGGPaqkcqGGBbWwcqWGfbqrdaWgaaWcbaGaeG4mamdabeaakiabc2faDbqaaiabdUealnaaBaaaleaacqaIZaWmcqaI0aanaeqaaOGaei4waSLaemiwaG1aaSbaaSqaaiabisda0aqabaGccqGGDbqxcqGHRaWkcqWGlbWsdaWgaaWcbaGaeG4mamJaeGOmaidabeaakiabcUfaBjabdIfaynaaBaaaleaacqaIZaWmaeqaaOGaeiyxa0Laey4kaSIaem4saS0aaSbaaSqaaiabiodaZiabisda0aqabaGccqWGlbWsdaWgaaWcbaGaeG4mamJaeGOmaidabeaaaaaaaa@A4C9@
3	d[X4]dt=(K31K32[X3]−K33K34[X4])[E3]K34[X4]+K32[X3]+K34K32−K41[X4][E4]K42+[X4]+K52[X6]−K51[X4][E2] MathType@MTEF@5@5@+=feaafiart1ev1aaatCvAUfKttLearuWrP9MDH5MBPbIqV92AaeXatLxBI9gBaebbnrfifHhDYfgasaacH8akY=wiFfYdH8Gipec8Eeeu0xXdbba9frFj0=OqFfea0dXdd9vqai=hGuQ8kuc9pgc9s8qqaq=dirpe0xb9q8qiLsFr0=vr0=vr0dc8meaabaqaciaacaGaaeqabaqabeGadaaakeaadaWcaaqaaiabdsgaKjabcUfaBjabdIfaynaaBaaaleaacqaI0aanaeqaaOGaeiyxa0fabaGaemizaqMaemiDaqhaaiabg2da9maalaaabaGaeiikaGIaem4saS0aaSbaaSqaaiabiodaZiabigdaXaqabaGccqWGlbWsdaWgaaWcbaGaeG4mamJaeGOmaidabeaakiabcUfaBjabdIfaynaaBaaaleaacqaIZaWmaeqaaOGaeiyxa0LaeyOeI0Iaem4saS0aaSbaaSqaaiabiodaZiabiodaZaqabaGccqWGlbWsdaWgaaWcbaGaeG4mamJaeGinaqdabeaakiabcUfaBjabdIfaynaaBaaaleaacqaI0aanaeqaaOGaeiyxa0LaeiykaKIaei4waSLaemyrau0aaSbaaSqaaiabiodaZaqabaGccqGGDbqxaeaacqWGlbWsdaWgaaWcbaGaeG4mamJaeGinaqdabeaakiabcUfaBjabdIfaynaaBaaaleaacqaI0aanaeqaaOGaeiyxa0Laey4kaSIaem4saS0aaSbaaSqaaiabiodaZiabikdaYaqabaGccqGGBbWwcqWGybawdaWgaaWcbaGaeG4mamdabeaakiabc2faDjabgUcaRiabdUealnaaBaaaleaacqaIZaWmcqaI0aanaeqaaOGaem4saS0aaSbaaSqaaiabiodaZiabikdaYaqabaaaaOGaeyOeI0YaaSaaaeaacqWGlbWsdaWgaaWcbaGaeGinaqJaeGymaedabeaakiabcUfaBjabdIfaynaaBaaaleaacqaI0aanaeqaaOGaeiyxa0Laei4waSLaemyrau0aaSbaaSqaaiabisda0aqabaGccqGGDbqxaeaacqWGlbWsdaWgaaWcbaGaeGinaqJaeGOmaidabeaakiabgUcaRiabcUfaBjabdIfaynaaBaaaleaacqaI0aanaeqaaOGaeiyxa0faaiabgUcaRiabdUealnaaBaaaleaacqaI1aqncqaIYaGmaeqaaOGaei4waSLaemiwaG1aaSbaaSqaaiabiAda2aqabaGccqGGDbqxcqGHsislcqWGlbWsdaWgaaWcbaGaeGynauJaeGymaedabeaakiabcUfaBjabdIfaynaaBaaaleaacqaI0aanaeqaaOGaeiyxa0Laei4waSLaemyrau0aaSbaaSqaaiabikdaYaqabaGccqGGDbqxaaa@9B12@
4	d[X5]dt=K41[X4][E4]K42+[X4] MathType@MTEF@5@5@+=feaafiart1ev1aaatCvAUfKttLearuWrP9MDH5MBPbIqV92AaeXatLxBI9gBaebbnrfifHhDYfgasaacH8akY=wiFfYdH8Gipec8Eeeu0xXdbba9frFj0=OqFfea0dXdd9vqai=hGuQ8kuc9pgc9s8qqaq=dirpe0xb9q8qiLsFr0=vr0=vr0dc8meaabaqaciaacaGaaeqabaqabeGadaaakeaadaWcaaqaaiabdsgaKjabcUfaBjabdIfaynaaBaaaleaacqaI1aqnaeqaaOGaeiyxa0fabaGaemizaqMaemiDaqhaaiabg2da9maalaaabaGaem4saS0aaSbaaSqaaiabisda0iabigdaXaqabaGccqGGBbWwcqWGybawdaWgaaWcbaGaeGinaqdabeaakiabc2faDjabcUfaBjabdweafnaaBaaaleaacqaI0aanaeqaaOGaeiyxa0fabaGaem4saS0aaSbaaSqaaiabisda0iabikdaYaqabaGccqGHRaWkcqGGBbWwcqWGybawdaWgaaWcbaGaeGinaqdabeaakiabc2faDbaaaaa@4CAF@
5	d[X6]dt=K51[X4][E2]−K52[X6]d[E2]dt=K52[X6]−K51[X4][E2] MathType@MTEF@5@5@+=feaafiart1ev1aaatCvAUfKttLearuWrP9MDH5MBPbIqV92AaeXatLxBI9gBaebbnrfifHhDYfgasaacH8akY=wiFfYdH8Gipec8Eeeu0xXdbba9frFj0=OqFfea0dXdd9vqai=hGuQ8kuc9pgc9s8qqaq=dirpe0xb9q8qiLsFr0=vr0=vr0dc8meaabaqaciaacaGaaeqabaqabeGadaaakqaabeqaamaalaaabaGaemizaqMaei4waSLaemiwaG1aaSbaaSqaaiabiAda2aqabaGccqGGDbqxaeaacqWGKbazcqWG0baDaaGaeyypa0Jaem4saS0aaSbaaSqaaiabiwda1iabigdaXaqabaGccqGGBbWwcqWGybawdaWgaaWcbaGaeGinaqdabeaakiabc2faDjabcUfaBjabdweafnaaBaaaleaacqaIYaGmaeqaaOGaeiyxa0LaeyOeI0Iaem4saS0aaSbaaSqaaiabiwda1iabikdaYaqabaGccqGGBbWwcqWGybawdaWgaaWcbaGaeGOnaydabeaakiabc2faDbqaamaalaaabaGaemizaqMaei4waSLaemyrau0aaSbaaSqaaiabikdaYaqabaGccqGGDbqxaeaacqWGKbazcqWG0baDaaGaeyypa0Jaem4saS0aaSbaaSqaaiabiwda1iabikdaYaqabaGccqGGBbWwcqWGybawdaWgaaWcbaGaeGOnaydabeaakiabc2faDjabgkHiTiabdUealnaaBaaaleaacqaI1aqncqaIXaqmaeqaaOGaei4waSLaemiwaG1aaSbaaSqaaiabisda0aqabaGccqGGDbqxcqGGBbWwcqWGfbqrdaWgaaWcbaGaeGOmaidabeaakiabc2faDbaaaa@6C8D@

**Table 2:** Kinetic parameters used in the model. These values were determined artificially. Details of the kinetic parameters are shown in Table 1. The search ranges were *K*_*i *_∈ [0.1,1000].

Parameter value	
*K*_11 _= 10	*K*_31 _= 1000
*K*_12 _= 0.1	*K*_32 _= 1
*K*_13 _= 4	*K*_33 _= 0.1
*K*_14 _= 3	*K*_34 _= 1
*K*_21 _= 1000	*K*_41 _= 2
*K*_22 _= 1	*K*_42 _= 1
*K*_23 _= 0.1	*K*_51 _= 1000
*K*_24 _= 3	*K*_52 _= 0.1

**Table 3:** Initial conditions of training data sets from No.1 to No.10 and test data sets from No.11 to No.20. Examples of time-courses using initial conditions No.3 and No.5 are shown in Figure 3. Concentrations are non-unit.

Training	*X*_1_	*X*_2_	*X*_3_	*X*_4_	*X*_5_	*X*_6_
1	4.5	0.4	0.3	3.4	0	4.2
2	1.7	0.5	0.8	1.0	0	4.3
3	4.2	0.4	0.7	3.3	0	1.2
4	4.1	0.3	0.2	4.1	0	2.1
5	1.3	1.5	1.8	1.6	0	0.2
6	4.2	3.9	0.1	0.2	0	0.1
7	4.2	0.1	4.6	0.2	0	4.1
8	0.3	0.7	0.3	3.5	0	2.0
9	4.5	0.5	0.6	4.1	0	0.3
10	4.1	0.2	0.1	2.5	0	4.2

Test	*X*_1_	*X*_2_	*X*_3_	*X*_4_	*X*_5_	*X*_6_

11	0.5	0.5	0.3	1.0	0	0.1
12	2.3	2.7	1.3	1.0	0	1.1
13	1.7	1.5	3.2	1.4	0	1.6
14	1.2	1.8	2.6	1.9	0	3.1
15	1.3	1.7	1.5	2.0	0	2.6
16	2.2	2.4	2.7	3.2	0	2.1
17	2.3	2.3	2.0	1.0	0	0.1
18	3.1	3.1	3.2	3.7	0	2.6
19	3.6	2.7	3.0	2.6	0	3.9
20	1.9	2.3	2.5	1.7	0	4.6

**Table 4:** Parameters applied in this work. The top column shows the parameters of RBFN, the bottom column the parameters of ADSGA using SPX as the recombination- and ranking selection as the selection procedure.

parameter of RBFN	meaning	value
*P*_*near*_	number of near individual	1
*P*_*far*_	number of far individual	1
*Lo*	initial *L*	0.4
α	neighborhood control parameter	0.02
λ	generalization control parameter	0.1
parameter of ADSGA	meaning	value

*P*_*GA*_	number of individual	30
*G*_*GA*_	max of generation	100
*m*_0_	initial mutation ratio	0.05
η^+^	selection control parameter	2.0
η^-^	selection control parameter	0.0
ε	extension rate	1.0

**Table 5:** Parameters for GA. SPX is employed as the recombination method, ranking selection as the selection method.

parameter	meaning	value
*P*_*GA*_	number of individual	30
*m*_0_	initial mutation ratio	0.05
η^+^	selection control parameter	2.0
**η **^-^	selection control parameter	0.0
**ε**	extension rate	1.1

**Table 6:** Validation of two subsidiary improvements and comparative experiments between GA and RBFN including above-mentioned improvements. The comparison between *k*NN and ADSGA are shown in the columns headed *k*NN and the second columns from right headed ADSGA. The results acquired with the conventional *k*NN method and our proposed ADSGA are shown in the columns headed *k*NN and ADSGA. The improved results obtained with the fitness transformation are demonstrated in the 2 columns headed ADSGA and ADSGA+Log. The validation of main improvement that is the application of RBFN is presented in the leftmost GA column and the rightmost ADSGA+Log column. The proposed method yielded equivalent or more accurate results compared to the parameters obtained with GA at half the calculation time and a 50% increase in the optimization success rate.

	GA	RBFN					
		*k*NN				ADSGA	ADSGA Log
		*k *= 2	*k *= 4	*k *= 8	*k *= 16		

Convergence rate (%)	60	90	88	92	90	86	90
Processing time (min)	273	163	188	170	157	167	130
Test error (%)	10.3 ± 2.5	12.9 ± 3.9	10.4 ± 2.0	11.0 ± 2.3	11.8 ± 3.4	9.3 ± 2.2	10.8 ± 2.1
